# Plasminogen Activator Inhibitor-1 Antagonist TM5484 Attenuates Demyelination and Axonal Degeneration in a Mice Model of Multiple Sclerosis

**DOI:** 10.1371/journal.pone.0124510

**Published:** 2015-04-27

**Authors:** Nicolas Pelisch, Takashi Dan, Atsuhiko Ichimura, Hiroki Sekiguchi, Douglas E. Vaughan, Charles van Ypersele de Strihou, Toshio Miyata

**Affiliations:** 1 Department of Molecular Medicine and Therapy, United Centers for Advanced Research and Translational Medicine, Tohoku University Graduate School of Medicine, Sendai, Miyagi, Japan; 2 Department of Medicine, Northwestern University Feinberg School of Medicine, Chicago, Illinois, United States of America; 3 Service de Nephrologie, Cliniques Universitaires Saint Luc, Brussels, Belgium; University of Muenster, GERMANY

## Abstract

Multiple sclerosis (MS) is characterized by inflammatory demyelination and deposition of fibrinogen in the central nervous system (CNS). Elevated levels of a critical inhibitor of the mammalian fibrinolitic system, plasminogen activator inhibitor 1 (PAI-1) have been demonstrated in human and animal models of MS. In experimental studies that resemble neuroinflammatory disease, PAI-1 deficient mice display preserved neurological structure and function compared to wild type mice, suggesting a link between the fibrinolytic pathway and MS. We previously identified a series of PAI-1 inhibitors on the basis of the 3-dimensional structure of PAI-1 and on virtual screening. These compounds have been reported to provide a number of *in vitro* and *in vivo* benefits but none was tested in CNS disease models because of their limited capacity to penetrate the blood-brain barrier (BBB). The existing candidates were therefore optimized to obtain CNS-penetrant compounds. We performed an *in vitro* screening using a model of BBB and were able to identify a novel, low molecular PAI-1 inhibitor, TM5484, with the highest penetration ratio among all other candidates. Next, we tested the effects on inflammation and demyelination in an experimental allergic encephalomyelitis mice model. Results were compared to either fingolimod or 6α-methylprednisolone. Oral administration of TM5484 from the onset of signs, ameliorates paralysis, attenuated demyelination, and axonal degeneration in the spinal cord of mice. Furthermore, it modulated the expression of brain-derived neurotrophic factor, which plays a protective role in neurons against various pathological insults, and choline acetyltransferase, a marker of neuronal density. Taken together, these results demonstrate the potential benefits of a novel PAI-1 inhibitor, TM5484, in the treatment of MS.

## Introduction

Multiple sclerosis (MS), a chronic, inflammatory, demyelinating disease of the central nervous system (CNS), is a leading cause of disability in young, mainly female, adults [1]. Its pathological hallmarks are inflammation and demyelination; they follow the entry of fibrinogen into the CNS, inducing an inflammatory response and axonal damage [2,3].

A link between MS and alterations in the endogenous fibrinolitic system has been identified previously. In particular, increased plasminogen activator inhibitor 1 (PAI-1) levels in the cerebrospinal fluid as well as in acute lesions of patients with MS have been described [4,5]. In the same line of evidence, PAI-1 deficient mice appear to be at least partially protected from chronic relapsing experimental allergic encephalomyelitis (CREAE), a model of MS [6].

Previously, we developed a series of orally active, low molecular PAI-1 inhibitors, relying on virtual screening and the 3-dimentional structure of the complex of PAI-1 with its inhibitory peptide [7]. In addition to their predictable anti-thrombotic effects, these compounds have demonstrated to be valuable in numerous preclinical models, including pulmonary fibrosis, macrophage infiltration, bone marrow regeneration, and arteriosclerosis [7,8,9,10]. However, none of these PAI-1 antagonists have been investigated in CNS diseases models because of their inability to cross the blood brain barrier (BBB). We therefore optimized the existing candidates in order to obtain a drug with CNS-penetrant properties.

Traditionally, a series of physicochemical properties, such as low molecular weight, high lipophilicity (clogP) as well as low polarity (TPSA), are needed to allow effective penetration into the CNS. We selected a class of PAI-1 inhibitors meeting these properties and analyzed their ability to cross the BBB using an *in vitro* model corresponding with the anatomical situation of cerebral microvessels [11]. Eventually, we identified, among all other candidates, a novel, small molecule PAI-1 inhibitor, TM5484, with the highest penetration ratio through the BBB.

We then explored its therapeutic effects on neuroinflammation, demyelination and axonal degeneration, using a mouse model of MS. The effects of the PAI-1 inhibitor were compared with either fingolimod or 6α-methylprednisolone, two drugs currently used to treat patients with MS [12,13]. In addition, we tested the possibility that TM5484 produces neuroprotection through the modulation of the brain-derived neurotrophic factor (BDNF), a member of the neurotrophin family of growth factors known to play a key role in neurons survival, and choline acetyltransferase (ChAT), a marker of neuronal density. A direct link between BDNF and some components of the fibrinolytic pathway has been documented previously [14], but very little is known about PAI-1 and BDNF in MS. Our results indicate that a small molecular PAI-1 inhibitor protects against neuroinflammation, demyelination and axonal degeneration in a mice model of MS, thus validating TM5484 as a potential therapeutic agent.

## Materials and Methods

### Reagents

Dimethyl sulfoxide (DMSO) was purchased from Nacalai Tesque (Kyoto, Japan), fingolimod (FTY720) from Selleck Chemicals (Houston, TX, USA), and 6α-methylprednisolone, propanolol and verapamil from Sigma Aldrich (Tokyo, Japan).

### TM5484 activity and specificity

TM5484 was developed, as a derivative of the PAI-1 inhibitor TM5441 [9], at the United Centers for Advanced Research and Translational Medicine (ART), Tohoku University Graduate School of Medicine, Miyagi, Japan. Its PAI-1 inhibitory activity and specificity were assessed by a chromogenic assay as previously described [7,8]. In brief, the reaction mixture includes 0.15 mol/L NaCl, 50 mmol/L Tris-HCl pH 8, 0.2mmol/L CHAPS, 0.1% PEG-6000, 1% dimethylsulfoxide, 5 nmol/L of either human active PAI-1 (Molecular Innovations, Southfield, MI), human antithrombin III (Sigma-Aldrich, Saint Louis, MO), or human α2-antiplasmin (Sigma-Aldrich), 2 nmol/L of either human 2-chain tPA (American Diagnostica Inc, Stanford, CT), thrombin (Sigma-Aldrich), or plasmin (Sigma-Aldrich), and 0.2 mmol/L of either Spectrozyme tPA (Chromogenix, Milano, Italy), chromogenic substrate S-2238 (Sekisui medical, Tokyo, Japan), or chromogenic substrate S-2251 (Sekisui medical) at a final concentration. Tested compounds were added at various concentrations and the half-maximal inhibition (IC50) was calculated by logit-log analysis. TM5484 inhibited PAI-1 activity with an IC50 value of 3.56 mM but did not inhibit α2-antiplasmin.

### TM5484 pharmacokinetics and toxicity

TM5484, suspended in a 0.5% carboxymethyl cellulose sodium salt solution, was administered orally by gavage feeding to male Wistar rats (5 mg/kg; CLEA Japan Inc.). Heparinized blood samples were collected from the vein before (0 h) and 1, 2, 6, and 24 h after oral drug administration. Plasma drug concentration was determined by reverse-phase high-performance liquid chromatography. Maximum drug concentration time (Tmax), maximum drug concentration (Cmax), and drug half-life (T1/2) were calculated as 2h, 22.5 μM and 1.7h, respectively. All toxicity studies followed the International Conference on Harmonization of Technical Requirements for Registration of Pharmaceuticals for Human Use Harmonized Tripartite Guidelines at the non-GLP conditions. A repeated-dose toxicity study of TM5484 (30 mg/kg) was conducted for 2 weeks in 5 Crl:CD (SD) male rats: no adverse effects were observed. TM5484 was negative for the reverse mutation Ames test. The effect of TM5484 on the human ether-a-go- go-related gene (hERG) electric current was investigated in HEL293 cells, transfected with the hERG gene: TM5484 had not effect on hERG electric current.

### Analysis of compounds penetration and concentration into CNS

In order to determine the ability of compounds to penetrate into CNS we used an *in vitro* model of BBB (RBT-24H, BBB Kit, PharmaCo-Cell Company Ltd. Nagasaki, Japan). Verapamil and Propanolol were used as a negative and positive control, respectively. This BBB model was reconstructed by the culture of both primary rat brain micro vascular endothelial cells and rat brain pericytes separated by a macroporous Millicell membrane (24 wells, pore size: 3.0 μm, Millipore, Bedford, MA, USA). Briefly, we pre-incubated the BBB kits at 37 °C in 5% CO_2_ conditions for 4–5 days, in order to strongly reconstruct the barrier integrity and tight-junctions. To evaluate the barrier integrity and confirm the functionality of the tight-junctions, trans-endothelial electrical resistance (TEER) was measured at day 5-post incubation. Assays were carried out using TEER values in the range of 150-to 300-Ω cm^2^ [11]. Once the tight junctions reach a TEER of 300-Ω cm^2^, 0.9 mL PBS-based assay medium was added to blank 24-well culture plates. Prior to incubation, selected compounds were diluted in DMSO 100%. The compounds suspended in 0.2 mL assay medium were fortified into the apical side and incubated for 30 min. Next, the medium from both the apical and the basolateral sides were collected and further analyzed by liquid chromatography–mass spectrometry. Transport was measured by detecting the amount of compound from the lower (basal, brain-side) compartment and was calculated using the apparent permeability coefficient (*P*app), a formula provided by PharmaCo-Cell Company Ltd.

### Experimental animals

All animal experiments conformed to the National Health Guide for the Care and Use of Laboratory Animals and were approved by the Animal Committee at Tohoku University. Studies were performed on 11-week-old female C57BL/6 mice (n = 8–10, each group) and 10-week-old Lewis rats (n = 6–8, each group), obtained from SJC, Japan SLC (Hamamatsu, Shizuoka, Japan). Animals were housed under a 12h light-dark cycle and given regular chow, MF (Oriental Yeast Co., Ltd.). At the onset of paralysis, TM5484 (5 mg/kg), fingolimod (0.5 mg/kg) or 6α-methylprednisolone (5 mg/kg) were given daily by gavage to mice diluted in Carmellose Sodium (Maruishi, Osaka, Japan) and ultra pure water.

### Induction and clinical evaluation of EAE

Experimental autoimmune encephalomyelitis (EAE) was induced in mice as described previously [15]. Briefly, on day 0, 200 microgramme of myelin oligodendrocyte glycoprotein^35–55^ (MOG^35–55^) peptide was injected s.c, twice, in the lower and upper back of the mice, in an equal volume of complete Freund’s adjuvant (Hooke Laboratories, Inc.). Next, 200 ng of pertussis toxin (Hooke Laboratories, Inc.) dissolved in PBS was applied i.p. On day 1, another 200 ng of pertussis toxin was administered. For rats, 200 microgramme of myelin basic protein^69-88^ (MBP^69-88^) was injected (Hooke Laboratories, Inc.) twice, in the lower back on day 0. Animals were scored for motor paralysis on a daily basis. Disease severity was assessed using a scale ranging from 0 to 5 (Hooke Laboratories, Inc.): 0, normal; 1, limp tail; 1.5, Limp tail and hind leg inhibition; 2, Limp tail and weakness of hind legs.; 2.5, Limp tail and dragging of hind legs; 3, Limp tail and complete paralysis of hind legs; 3.5, Limp tail and complete paralysis of hind legs and Hind legs are together on one side of body; 4, Limp tail, complete hind leg and partial front leg paralysis; 4.5, Complete hind and partial front leg paralysis, no movement around the cage; 5, Mouse is spontaneously rolling in the cage. Mice were typically observed for 28 days, during which they will remain chronically paralyzed. Rats were observed for three weeks: all groups including vehicle fully recovered, without relapses.

### Quantitative real-time polymerase-chain reaction (qRT-PCR)

Spinal cord was harvested from mice and snap frozen in liquid nitrogen (n = 6–7, each group). Total RNA was extracted from spinal cord and spleen using ISOGEN (Nippon Gene). qRT-PCR analysis was performed as described previously [10]. Briefly, qRT-PCR was performed on a LightCycler rapid thermal cycler system using a LightCycler 480 SYBR Green I Master (Roche Applied Science) according to the manufacturer's instructions. Data were analyzed by using the comparative Ct method as means of relative quantification, normalized to an endogenous reference (b-actin, Actb). The sequences of the primers are described below:

Mice Serpine sense: GATGGCTCAGACCAACAAGTTCAA

Mice Serpine antisense: TGGTAGGGCAGTTCCAGGATG

Mice Tnf sense: CTCCAGGCGGTGCTTGTTCC

Mice Tnf antisense: GGCTTGTCACTCGGGGTTCG

Mice Cd11b sense: ATAGTGACATTGCCTTCTTG

Mice Cd11b antisense: ATCTTGGGTTAGGGTTGTTC

Mice Il1b sense: AAATACCTGTGGCCTTGGGC

Mice Il1b antisense: CTTGGGATCCACACTCTCCAG

Mice Il6 sense: CACAGACAGCCACTCACCTC

Mice Il6 antisense: TCCAAAAGACCAGTGATGAT

Mice Il10 sense: CATGGCCCAGAAATCAAGGA

Mice Il10 antisense: GGAGAAATCGATGACAGCGC

Mice Cd68 sense: AGTGGACATTCTCGGCTCAG

Mice Cd68 antisense: ATGATGAGAGGCAGCAAGAT

Mice BDNF sense: ACGAAGAAAACCATAAGGAC

Mice BDNF antisense: CCACTCGCTAATACTGTCAC

Mice ChAT sense: TCATTAATTTCCGCCGTCTC

Mice ChAT antisense: AGTCCCGGTTGGTGGAGTC

Mice Ccl2 sense: CCTTCTGTGCCTGCTGCTCA

Mice Ccl2 antisense: ACTTGCTGCTGGTGATTCTT

Mice Il17 sense: CAGCAGCGATCATCCCTCAAAG

Mice Il17 antisense: CAGGACCAGGATCTCTTTGCTG

Mice IFNγ sense: TCTGTCTCCTCAACTATTTC

Mice IFNγ antisense: CATCTCCTCCCATCAGCAGC

Mice Actb sense: TGGCACCCAGCACAATGAA

Mice Actb antisense: CTAAGTCATAGTCCGCCTAGAAGCA

### Histology

Mice were perfused in the heart with saline 0.9% followed by neutral-buffered formalin 10% (n = 3-4/group). Slides from different sections of the spinal cord were immersed and fixed overnight in 10% neutral-buffered formalin. Next, samples were transfered and immersed in a 10, 20 and 30% sucrose solution for consecutive nights. Sections were embedded in O.C.T compounds (Sakura Finetek, Japan) for the subsequent immunostaining study. Four sections of the spinal cord from each group were examined. Since the clinical manifestations are typically initiated as limp tail and hind-limb paralysis in this model, we focused on lumbar spinal segments. Frozen specimens were then incubated overnight at room temperature and stained with Cd11b for macrophage infiltration (Abcam), myelin basic protein (MBP) for demyelination, neurofilament non-phosphorylated (SMI-32) for axonal damage, collagen IV for extracellular matrix depositions (Abcam) and fibrinogen (Funakoshi, Japan). Incubation for 2 hs at room temperature in corresponding biotinylated goat secondary antibody was performed. For immunofluorescence, the sections were incubated overnight at room temperature using primary antibody Iba-1 for microglia activation (Wako Chemicals). After wash in PBS, the sections were then incubated in corresponding fluorescent goat secondary antibody (Wako Chemicals). For Cd11b, the number of positive macrophage was quantified as the total cells per selected area. The area of demyelination was calculated as the percentage of negative staining for MBP *per* selected area. As for axonal damage and collagen IV deposition, the positive staining *per* selected area was analyzed and calculated as the percentage *per* total area. For Quantification analysis was assessed by ImageJ TM software (version 1.440, National Institute of Health, Bethesda, MD, USA).

### Foot print analysis

The footprint test was used to measure and compare the stride length (the mean distance of forward movement between each stride) of EAE mice with that of TM5484 and fingolimod treated mice, as previously described [16]. Briefly, to obtain footprints, the hind- and forefeet of the mice were coated with black non-toxic paint. The floor of the open field box was covered with white paper. Each mouse was placed in a corner of the open field and allowed to walk across the paper, leaving a track of footprints. If a mouse didn’t walk forward, the task was considered a failure. For each step parameter, three values were measured from each run, excluding footprints made at the beginning and end of the run where the animal was initiating and finishing movement respectively. The mean value of each set of three values was used for analysis.

### Statistical analysis

The significance of the difference between data sets was assessed by the Student’s t-test. Differences between multiple groups were analyzed by one-way ANOVA test followed by the Tukey HSD or Dunnett’s test for comparison between treatment groups. All statistical analyses were performed using the Prism software (version 5.0, GraphPad Software, La Jolla, CA, USA). *In vitro* data and *in vivo* data were expressed as means ± standard error and means ± standard division, respectively. P<0.05 was considered to be statistically significant.

## Results

### TM5484 is a PAI-1 inhibitory compound highly penetrating into CNS

**Fig 1:**
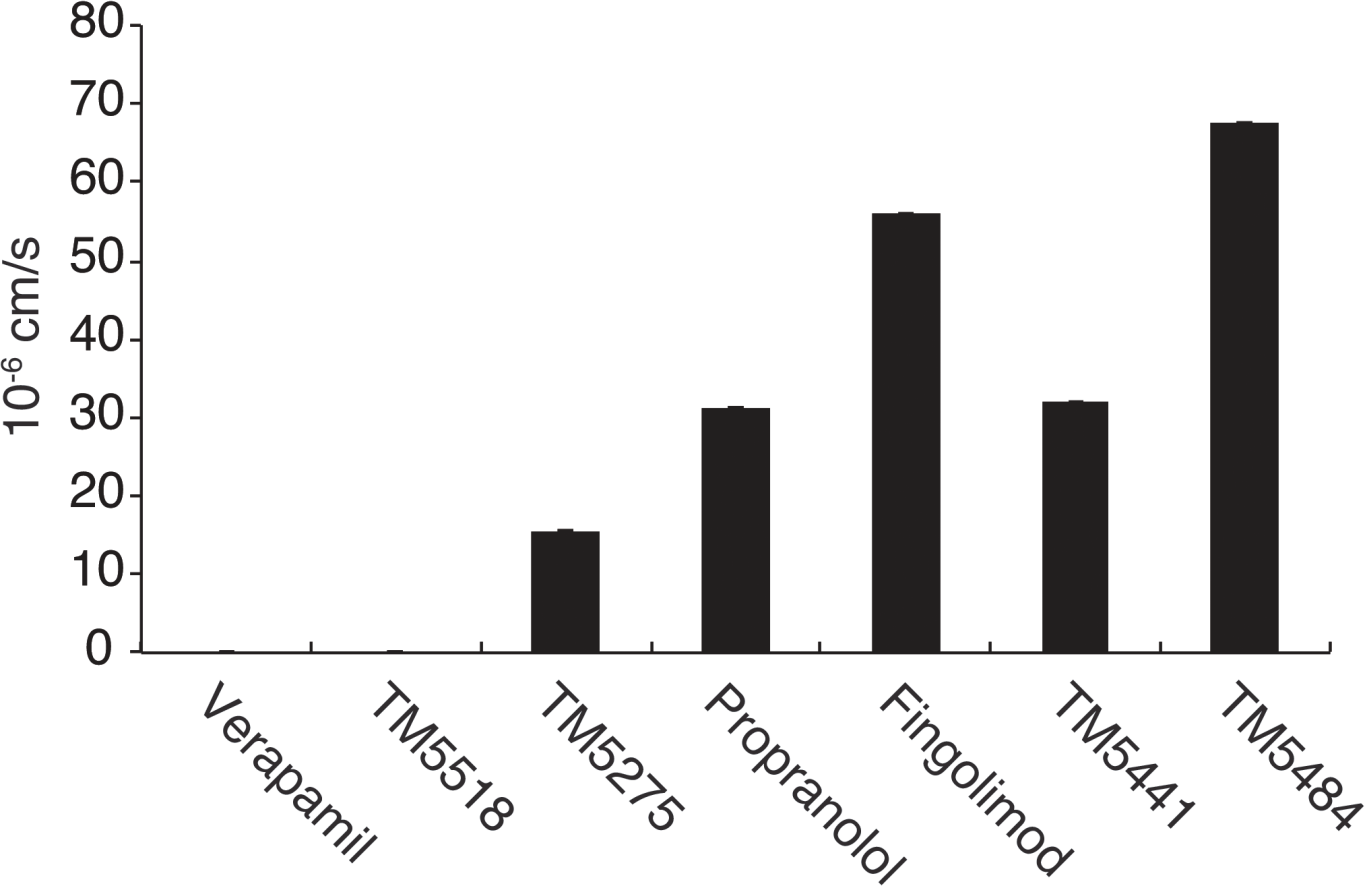
*In vitro* BBB penetration assay. Analysis of the penetration ratio of different compounds using an *in vitro* model of BBB. A permeability coefficient (Papp) above 20 x 10^–6^ cm/s is considered to provide a very good penetration into the CNS, while a Papp lower than 10 x 10^–6^ cm/s is consistant with a low penetrant CNS drug. TM5484 has a 67.6 x 10^–6^ cm/s permeability coefficient. Verapamil (N.D.) and Propanolol (31.3 x 10^–6^ cm/s) were used as negative and positive control, respectively. Data are mean±SD. n = 3 for each compound. N.D. indicates not detectable.

We synthesized over 500 derivatives of the lead compound TM5275 and systematically tested the compounds to identify a candidate able to cross the BBB (S1 Fig). Taking into account the requirements for a good CNS penetration [17], we selected about 50 compounds with a low MW, a lipophilicity (clogP) < 4 and a surface area (TPSA) > 75. As shown in Fig 1, a drug with a Papp above 20 x 10^–6^ cm/s was considered to have a very good penetration into the CNS, while a compound with a Papp lower than 10 x 10^–6^ cm/s was considered a low penetrant CNS drug. As expected, fingolimod, with a Papp of 56 x 10^–6^ cm/s, was found at the brain side of the insert. Propanolol, a sympatholytic non-selective beta-blocker, had a Papp of 31.3 x 10^–6^ cm/s. TM5441 (M.W., 428.8; clogP, 2.39; TPSA, 117.9), had a Papp of 32.1 x 10^–6^ cm/s. Further structural optimization of TM5441 generated TM5484 (M.W., 384.7; clogP, 3.07; TPSA, 108.6) with a Papp of 67.6 x 10^–6^ cm/s, indicating the highest penetration across the BBB.

### TM5484 attenuates paralysis and motor coordination in a mouse model of MS

**Fig 2:**
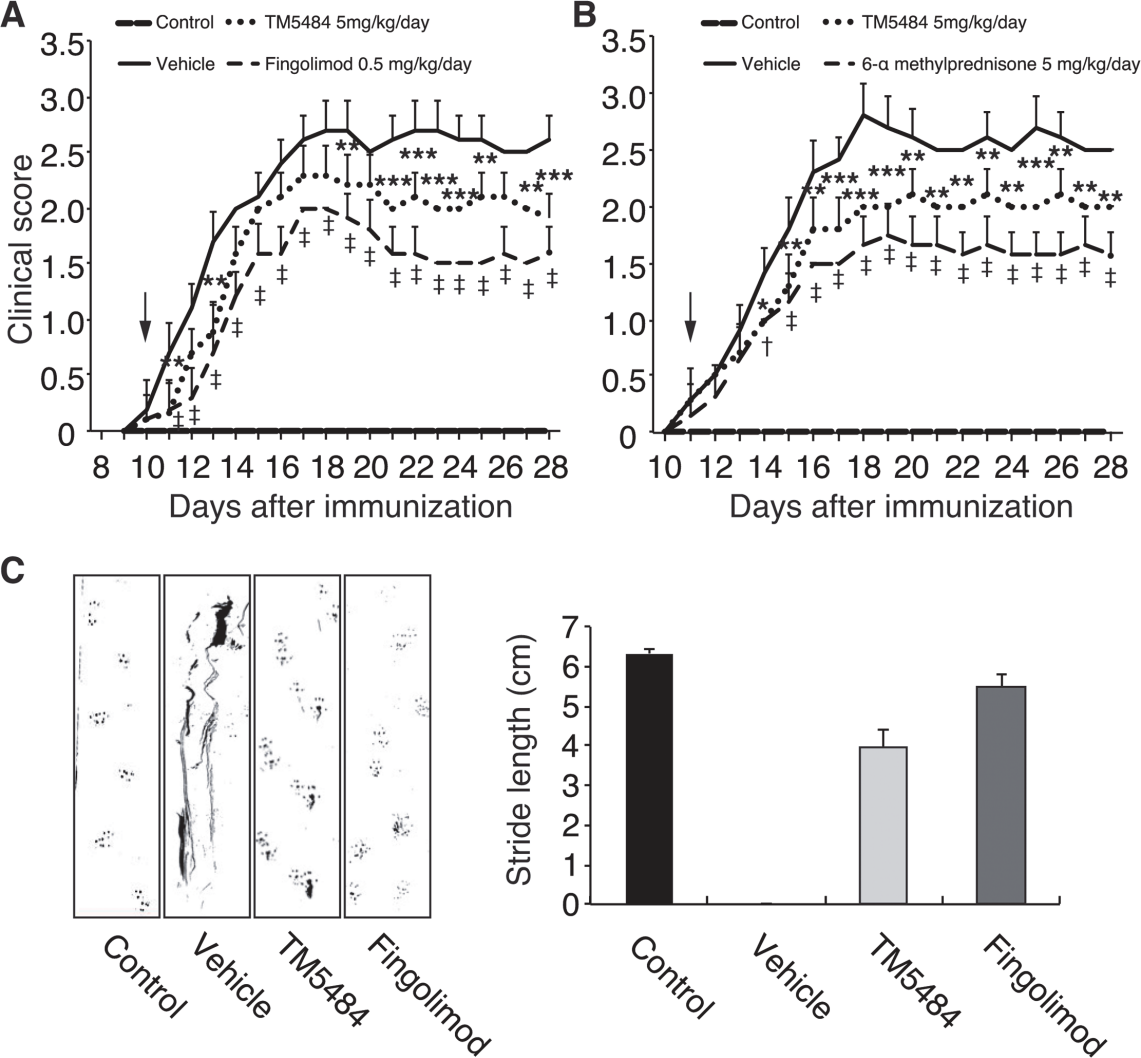
Clinical score and foot print assay. Mice were injected with myelin oligodendrocyte glycoprotein^35-55^ peptide in an equal volume of complete Freund’s adjuvant. TM5484 (5 mg/kg/d) given from the onset of symptoms significantly reduced the paralysis score compared to EAE untreated mice. Fingolimod (A, 0.5 mg/kg) and 6-α methylprednisone (B, 5 mg/kg/d) attenuated the paralysis score to the level observed with TM5484. C, TM5484 attenuates EAE-induced motor coordination impairment. Each mouse was placed in a corner of the open field and allowed to walk across the paper, leaving a track of footprints. For each step parameter, footprints made at the beginning and end of the run were excluded. Stride length of EAE untreated mice was considered 0 cm due to dragging. TM5484 treated mice had a stride length of 4.12 cm at dpi. 28, while Fingolimod treated animals showed a stride length of 5.48. The mean value of each set of three values was used for analysis. Data are shown as the means and corresponding SD. **P<0.01 or ***P<0.001 Vehicle *vs* TM5484, †*P* < 0.01, ‡*P* < 0.001 Vehicle *vs* Fingolimod or 6-α methylprednisone by 2-way ANOVA and Bonferroni test, n = 8–10.

EAE animals developed motor paralysis starting on day 10 or 11-post EAE induction (dpi) (Fig 2A and 2B). Mice given TM5484 at 5 mg/kg/d from the onset of signs had a significant amelioration of paralysis at the end of the experiment. In preliminary experiments we discovered that 5 mg/kg for TM5484 was the minimum effective dose for mice. Fingolimod (0.5 mg/kg) and 6α-methylprednisolone (5 mg/kg) also ameliorated the motor paralysis as compared to EAE group (Fig 2A and 2B). Dosage of these two drugs was chosen on the basis of previous studies [18,19].

We also assessed the effects of TM5484 on the impaired motor coordination caused by EAE induction. Mice were subjected to footprint analysis to assess the stride length. We found that EAE untreated mice had shorter stride lengths as compared to controls (Fig 2C). Most of this EAE untreated mice exhibited a dragging pattern due to severe paralysis of the hind legs. Oral administration of TM5484 as well as fingolimod significantly preserved the stride length of EAE mice.

### TM5484 mitigates inflammation, modulates BDNF and ChAT

**Fig 3:**
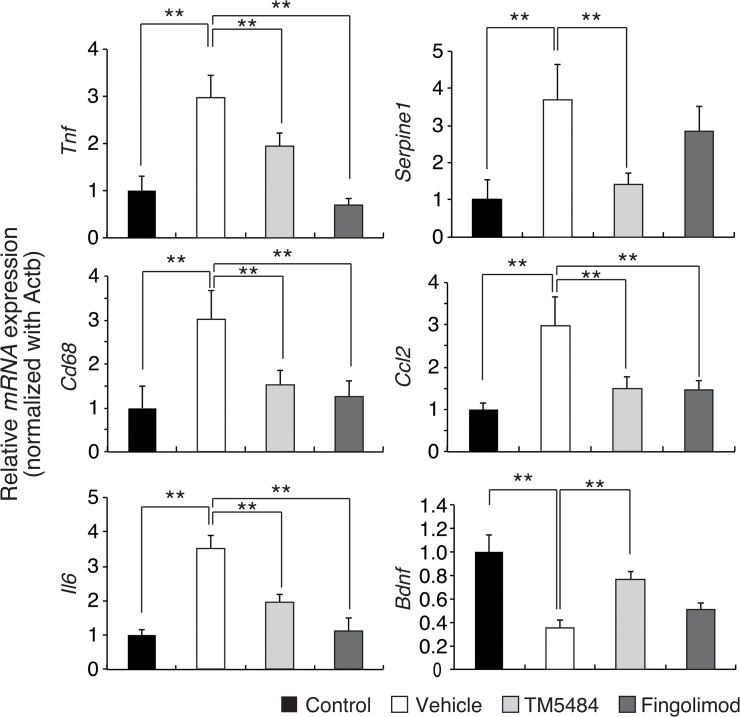
Gene expressions. Reverse transcriptase-polymerase chain reaction (RT-PCR) analysis of Tnf-1, PAI-1, Cd68, Ccl2, il-6 and BDNF in spinal cord. EAE mice show an increased expression of proinflammatory cytokines as well as a decreased expression of BDNF in spinal cord. Fingolimod has no effects on expressions of PAI-1 and BDNF. Expression levels of all markers are normalized to b-actin. Data are shown as the means and corresponding SEM. **P<0.01 by 1-way ANOVA and Dunnett test, n = 6–7

Previous studies reported that PAI-1 not only promotes leukocyte adhesion and migration but also plays a role in the inflammatory response and cell infiltration in MS [20,21,22]. We therefore investigated the potential effects of TM5484 on these processes in the CNS as well as in the periphery, using quantitative RT-PCR. As expected, gene expression of tumor necrosis alpha (TNF-α), interleukin-6 (IL-6) and chemokine Ligand-2 (Ccl2) were increased in EAE untreated mice, measured at dpi. 28 (Fig 3 and S2 Fig). Cd68, a marker of microglia activation, was also elevated. TM5484 or fingolimod, started from the onset of paralysis, provided similar reductions on these inflammatory markers in the spinal cord and spleen. Expression of IL-10, an antiinflammatory cytokine, was upregulated by TM5484 and fingolimod (S2 Fig). Elevation of PAI-1 mRNA expression in EAE mice was also prevented by TM5484.

We next examined whether TM5484 could also preserve brain tissue. In line with previous studies [23,24], BDNF as well as ChAT expression were significantly reduced in the spinal cord of EAE mice (Fig 3, panel at the bottom of the right column and S3 Fig). Importantly, TM5484 treatment was able to up-regulate levels of BDNF and ChAT similar to those of control group. In contrast to previous reports [25], fingolimod did not restore BDNF expression in the spinal cord of EAE mice.

### TM5484 ameliorates demyelination, axonal degeneration and collagen IV deposition

**Fig 4:**
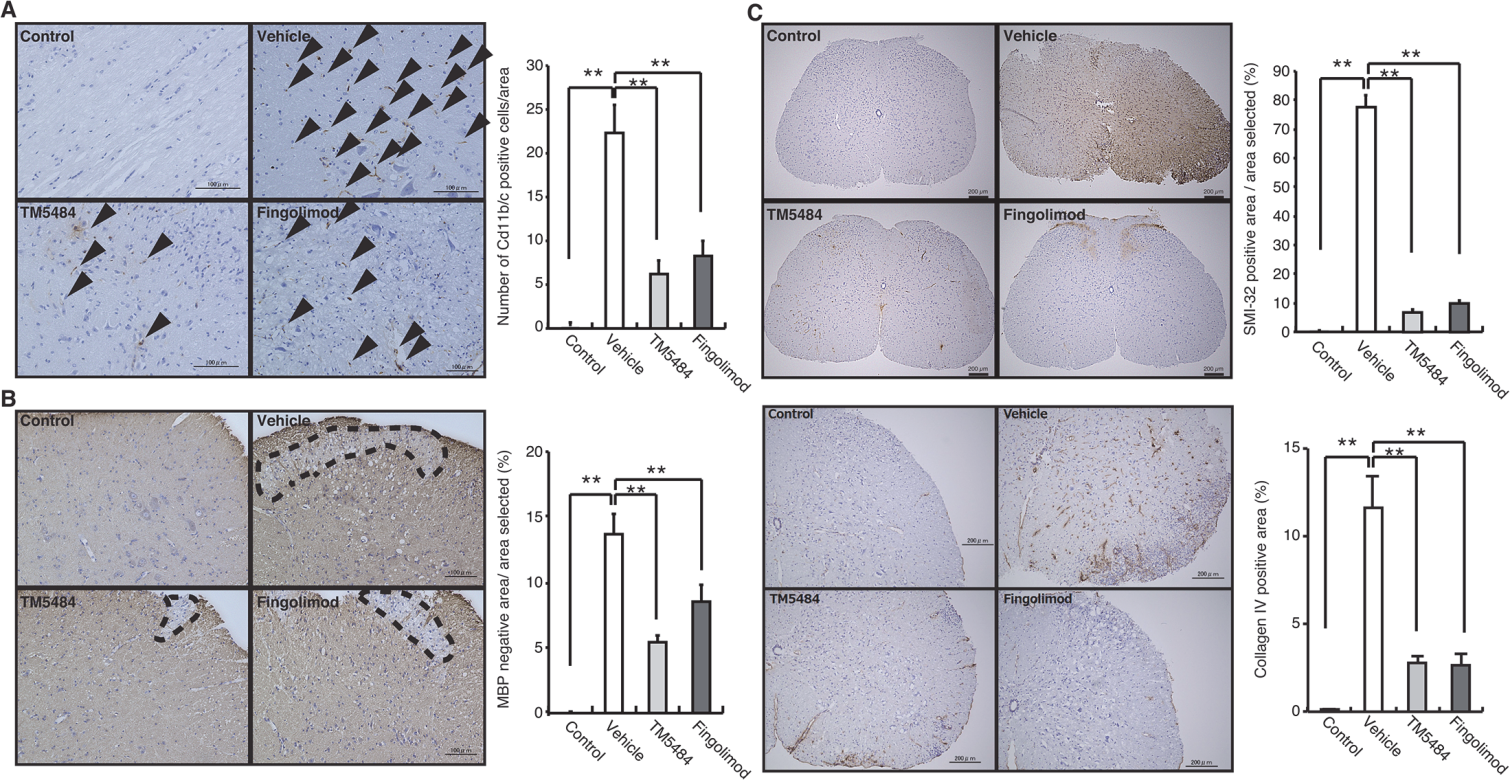
Histological analysis. Representative photomicrographs of control, vehicle, TM5484 (5 mg/kg/d) and fingolimod (0.5 mg/kg/d) sections at dpi. 28. Spinal cord was sectioned and stained with Cd11b (A), MBP (B), SMI-32 (C) and Collagen IV (D). Four sections of the spinal cord (lumbar spinal segments) from each group were examined. Data are expressed as the mean and corresponding SEM. TM5484 significantly reduced macrophage infiltration/microglia activation, demyelination, axonal degeneration as well as deposition of collagen IV in the spinal cord.

Extensive accumulation of macrophage and microglia activation in the spinal cord of EAE mice was demonstrated by increased Cd11b and Iba-1 staining (Fig 4A and S4 Fig). However, this was significantly reduced by oral administration of TM5484 and fingolimod.

Anti-MBP staining in spinal cord was then used to ascertain the effects of TM5484 on demyelination. Unsurprisingly, representative cross sections of the spinal cord revealed a higher degree of demyelination in EAE mice than in the control group (Fig 4B). However, this loss of myelin was attenuated by TM5484. In fingolimod treated mice, demyelination was also reduced. These results demonstrate that PAI-1 inhibitor TM5484 reduces the foci of demyelination and cell infiltration in the spinal cord of EAE mice. Interestingly, previous *in vivo* studies have demonstrated a similar attenuation of demyelination in the spinal cord of PAI-1 KO mice [6].

To test whether TM5484 could also protect brain tissue by preserving axons from damage, we performed histopathological analysis of the spinal cord with SMI-32, a marker of non-phosphorylated neurofilament indicating axonal damage. Increased amount of axonal degeneration was noted in the spinal cord of EAE mice at dpi 28 (Fig 4C). Yet, this was ameliorated by TM5484 treatment to an extent similar to that observed in the fingolimod-treated mice. The causes and pathogenesis of axonal degeneration, a feature common to many neurological disorders are still obscure, but a growing body of information indicates that both inflammation and demyelination play a major role; more specifically, microglia and macrophages, the main effectors of the innate immune response in the CNS, might be involved in axonal damage [26,27]. Taken together, these results suggest that PAI-1 inhibitor TM5484 could ameliorate axonal damage probably by attenuation of the immune response. Next, we also analyzed basement membrane proteins in the spinal cord. Deposition of collagen IV was increased in EAE untreated mice (Fig 4D). Both TM5484 and fingolimod however, were able to ameliorate collagen IV deposition in the spinal cord. This data highlights the possible changes of the extracellular matrix that occur in MS. Finally, fibrin deposition in spinal cord of EAE mice was also performed. TM5484 did not affect on the amount of fibrin deposition (S5 Fig), suggesting that PAI-1 enhanced by EAE might affect on the infiltration of inflammatory cells to a higher degree than extra cellular matrix components.

### TM5484 delays the onset of paralysis and has additive benefit on fingolimod

**Fig 5:**
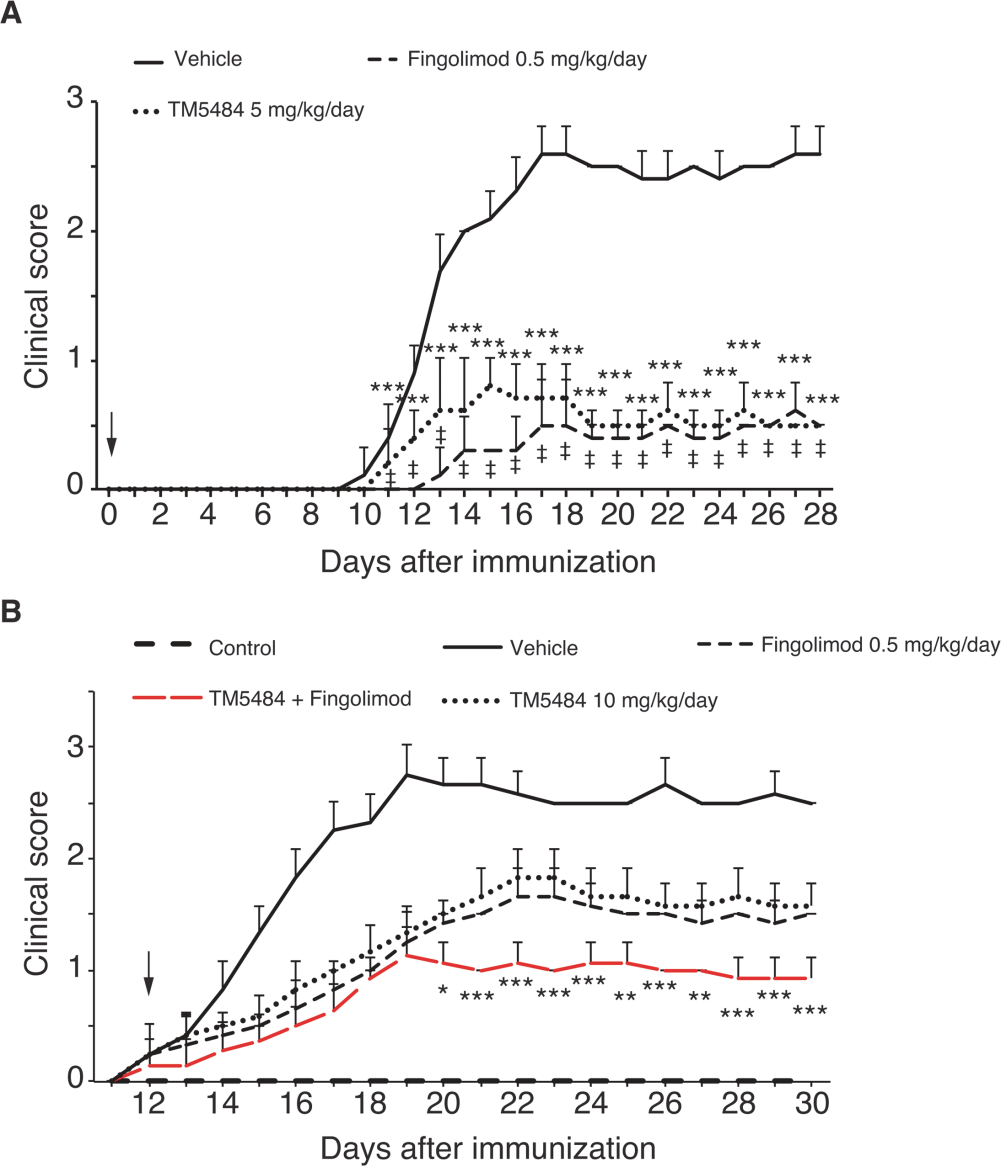
Clinical score (Preventive and combined therapy). Mice were injected with myelin oligodendrocyte glycoprotein^35-55^ peptide in an equal volume of complete Freund’s adjuvant. A, Administration of TM5484 (5 mg/kg/d) from day 0 not only abolished paralysis but also delayed the onset of symptoms in EAE mice. **P<0.01 or ***P<0.001 Vehicle *vs* TM5484, †*P* < 0.01, ‡*P* < 0.001 Vehicle *vs* Fingolimod by 2-way ANOVA and Bonferroni test, n = 8–10. B, Simultaneous administration of TM5484 (10 mg/kg/d) with fingolimod (0.5 mg/kg/d) attenuates the clinical score to a significantly greater extent than either TM5484 or fingolimod alone. Data are shown as the means and corresponding SEM. *P<0.05 or ***P<0.001 Fingolimod *vs* TM5484 + Fingolimod, by 2-way ANOVA and Bonferroni test, n = 8–10.

When administered from dpi 0 as a preventive treatment, TM5484 not only attenuated motor paralysis to a minimum score but also delayed the onset of signs in EAE mice (Fig 5A). Next, we analyzed a possible additive effect of TM5484 to fingolimod. As 0.5 mg/kg is the maximum therapeutic dose for fingolimod, we decided to use 10 mg/kg for TM5484 (the maximum therapeutic dose corroborated in our preliminary studies). Addition of TM5484 10 mg/kg to fingolimod, from the onset of signs in a combined treatment, attenuated the paralysis score to a greater extent than either drug alone (Fig 5B). Similarities to previously reported *in vivo* studies using t-PA as well as PAI-1 deficient mice [3,6] indicate that the beneficial effects of PAI-1 inhibitor TM5484 against paralysis in EAE mice are similar to the genetic knockout mice.

### TM5484 attenuates paralysis score in the rat MS model

**Fig 6:**
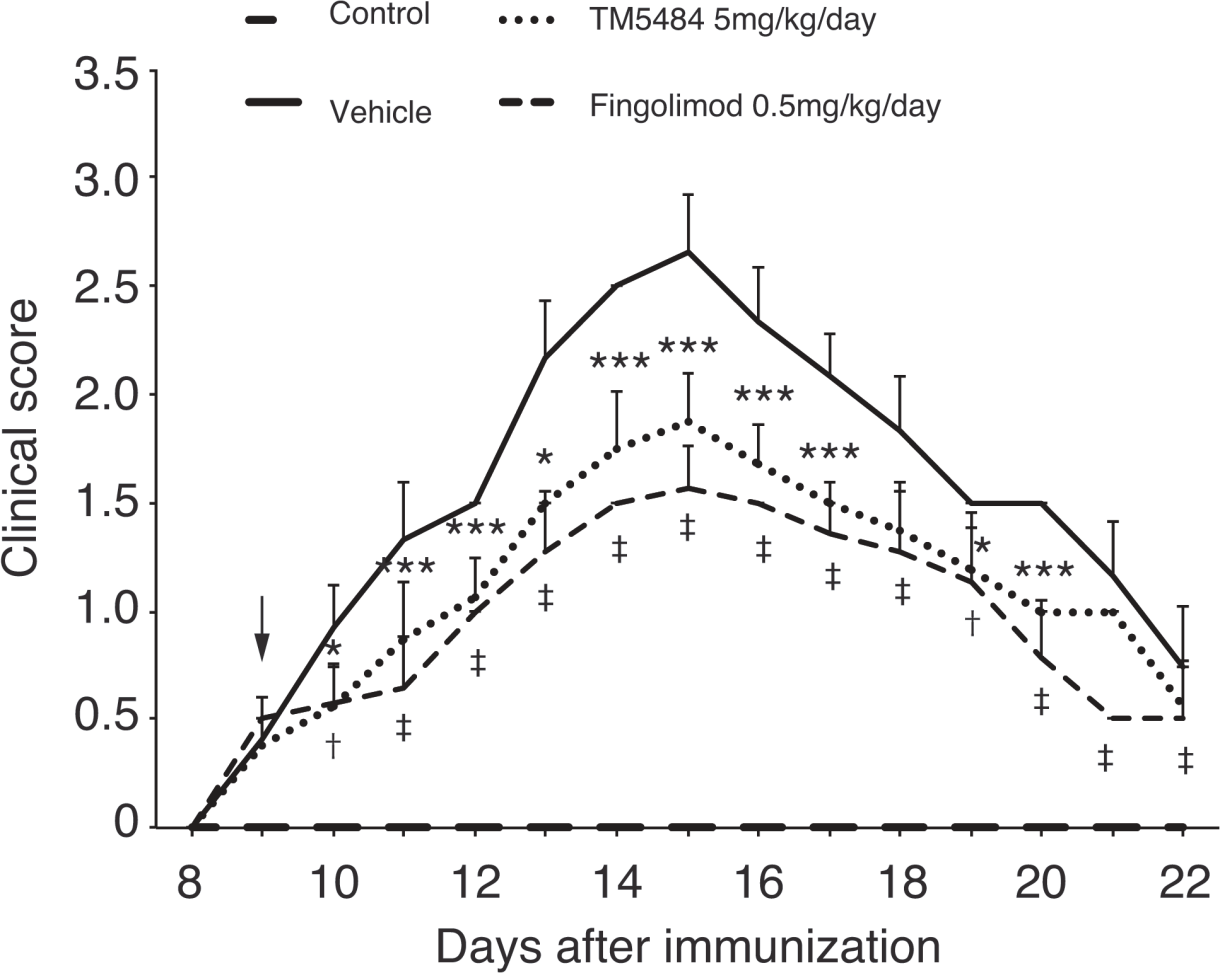
Clinical score in the rat MS model. Rats were injected with myelin basic protein^69-88^ peptide in an equal volume of complete Freund’s adjuvant. Administration of TM5484 (5 mg/kg/d) from the onset of symptoms significantly attenuated the paralysis score in EAE-treated rats (P<0.001 vs. vehicle at dpi. 15). Fingolimod (0.5 mg/kg) also reduced the paralysis score to a level similar to TM5484 (P<0.001 vs. vehicle at dpi. 15). The peak of the disease was selected to analyze the paralysis score as all animals, including vehicle, had an almost complete recovery by day 22. Data are shown as the means and corresponding SD. *P<0.05 or ***P<0.001 Vehicle *vs* TM5484, †*P* < 0.01, ‡*P* < 0.001 Vehicle *vs* Fingolimod by 2-way ANOVA and Bonferroni test, n = 6–8.

TM5484 treatment was also investigated in a rat model of EAE. Although the Lewis rat EAE may not be considered to be a demyelinating model, we have also used it to test TM5484, as some compounds exert a beneficial effect on mice but not in rats. Because all animals fully recovered by dpi. 21, the potential benefits of the drugs were measured at the peak of the paralysis (Fig 6). TM5484 (5 mg/kg/d), initiated at the onset of signs, was associated with a significant reduction of the paralysis score below that of EAE rats, in a similar manner seen with fingolimod treatment.

## Discussion

MS is the most common autoimmune inflammatory, demyelinating disease of the CNS [1]. Is often clinically punctuated by attacks and remissions with slowly progressive disability. Based upon the relation between PAI-1 and MS progression, we investigated the effects of a low molecular PAI-1 inhibitor, TM5484, and found that significantly attenuated neurological dysfunction in a mice model of MS as measured by the paralysis score.

Access to the CNS is essential to permit the effects of most neurotherapeutics. Lipophilicity and polar surface area are the most relevant determinants influencing brain penetration [28]. In order to improve the low CNS penetrance of our previous series of PAI-1 inhibitors, we systematically optimized their molecular structure and tested them in an *in vitro* model of BBB. Eventually, we found that PAI-1 inhibitors TM5441 and TM5484 exhibited the physicochemical properties needed by CNS-penetrant drugs. Because TM5484 demonstrated the highest penetration ratio, we focused on this compound in the present study.

TM5484 effectively suppressed the expression of proinflammatory cytokines implicated in MS and other neurodegenerative disorders [29]. Simultaneously, focal demyelination, axonal degeneration and collagen IV deposition decreased. The effects of TM5484 were not limited to the CNS, as it also down-regulated the expression of cytokines and up-regulated Il-10 in spleen of EAE mice, showing a modulation of the immune system in the periphery as well. Like this, it could be possible that TM5484 not only ameliorates the activation of resident microglia within the CNS but also prevents the migration of macrophage from periphery to the CNS.

Although it has been shown that pre treatment with a peptide derived from the PAI-1 protein inhibit EAE by immunosuppression [30], at present there is still a lack of evidence on how pharmacological inhibition of PAI-1 could reduce the loss of myelin and diminish the axonal degeneration typically seen during the course of EAE. The heterogeneous distribution of axonal damage in spinal cord, illustrated in our histological analysis, can also be seen when active demyelination occur and is consistent with a causal relationship between inflammation and axonal injury. Axonal damage is also likely to be multifactorial as numerous mediators of the cellular immune response are capable to damage axons [31]. Activated macrophages and microglia release nitric oxide, which causes reversible conduction block in demyelinated axons [32]. Attenuation of inflammation by TM5484 might thus protect axons by impeding the release of toxic soluble mediators.

Collagen IV is restricted to vascular basement membrane in the CNS of normal adults [33]; the expression of basement membrane components is altered in MS. Although is unclear how TM5484 ameliorates collagen IV deposition in CNS of EAE mice, it could be also related to its anti-inflammatory effects. For instance, van Horssen and colleagues showed that excessive deposition of collagen IV in CNS of patients with MS might be caused by increased production of cytokines [34]. TM5484 did not affect on the amount of fibrin deposition in spinal cord, suggesting that PAI-1-enhanced by EAE might affect on the infiltration of inflammatory cells to a higher degree than extra cellular matrix components.

There are a number of ways in which PAI-1 may contribute to the development of inflammation and demyelination in the CNS. For instance, PAI-1 can act as a chemoattractant for macrophages [10]. In the present study, gene expression and histological analyses consistently indicated that TM5484 treatment attenuated macrophage migration and microglia activation into the spinal cord, thereby attenuating demyelination and axonal damage.

Neurotrophic factors are known to protect neurons against various pathological insults. For instance, BDNF is a growth factor that plays important roles in the development and maintenance of the nervous system and induces neuronal survival [35]. In this study, EAE mice exhibited decreased mRNA expression of BDNF compared to control animals, while TM5484 treatment its BDNF expression. Inhibition of PAI-1 is predictably associated with a reciprocal increase in the activity of its target serine protease, t-PA, with secondary increases in net plasmin generation. Both of these serine proteases are known to convert secreted proBDNF to mature BDNF in the synaptic cleft [14]. In addition to its effects on demyelination and axonal degeneration, TM5484 may also accelerate neuronal repair in diseases linked to decreased BDNF levels. Although we only found differences of BDNF expression at the gene level, these findings suggest that TM5484, directly or indirectly modulate BDNF expression in neurons and thus might provide an additional mechanism for neuroprotection. To reinforce this, TM5484 also up-regulated the expression of ChAT, meaning a reduction of neuronal loss in spinal cord of EAE mice. This suggests that the effect of PAI-1 inhibitor TM5484 goes beyond blockage of inflammation, as it also induces prevention of damage and preservation of neuronal tissue. In contrast to previous studies using a mice model of Rett syndrome [36], our data showed that fingolimod did not restore the expression of mRNA BDNF in EAE mice.

Finally, the observed benefits of TM5484 were extended experimentally in rats. Admitting that the paralytic behavior seen in EAE rats is usually induced by infiltrating cells as well as by edema caused by blood-brain barrier disruption rather than by demyelination [37], when TM5484 was initiated at the time of onset of signs, motor paralysis was reduced to levels observed in untreated control, indicating that TM5484 also benefits the rat model of MS.

The mechanistic understanding of MS has advanced considerably over the past decade and has provided the rationale for the application of anti-inflammatory and immunomodulatory treatments that can actually reduce the severity and frequency of new demyelinating episodes [38]. Since TM5484 appears to influence several important pathogenic mechanisms in MS, it merit further investigation and consideration as a novel therapeutic modality for the treatment of MS.

In conclusion, we report evidence in this study that inhibition of PAI-1 with a low-molecular compound protect mice against EAE-induced inflammation, demyelination and axonal degeneration. TM5484 represents thus a novel therapeutic approach for MS and, perhaps, other CNS disorders.

## Supporting Information

S1 Fig Flow-chart of the identification of PAI-1 inhibitors efficiently penetrating thru the BBB. Based on virtual screening and *in silico* docking simulation we first developed a new group of oral, low molecular PAI-1 inhibitors. Among over 500 derivatives of the lead compound TM5275 we searched for a PAI-1 inhibitor that could efficiently penetrate into BBB. About 50 compounds, which met the criteria for a good CNS penetration, *i*.*e*., a low MW with a lipophilicity (clogP) < 4 and a surface area (TPSA) > 75, were selected and tested for CNS penetration, using an *in vitro* model that corresponds to the anatomical situation of cerebral microvessels. The penetration ratio (Papp) was finally measured. TM5484 (M.W., 384.7; clogP, 3.07; TPSA, 108.6), a derivative of TM5441 (Boe *et al* 2013), exhibited the highest penetration ratio across the BBB (Papp of 67.6 x 10^–6^ cm/s). It was selected for subsequent experiments.
(EPS)

S2 Fig Gene expression. Reverse transcriptase-polymerase chain reaction (RT-PCR) analysis of Tnf-1, IL-1b, IL-6, IL-10, IL-17 and IFNγ in spleen. EAE mice show an increased expression of proinflammatory cytokines as well as a decreased expression of IL-10. TM5484 and fingolimod were able to modulate these inflammatory effects. Expression levels of all markers are normalized to b-actin. Data are shown as the means and corresponding SEM. **P<0.01 by 1-way ANOVA and Dunnett test, n = 6–7
(EPS)

S3 Fig Gene expression. Reverse transcriptase-polymerase chain reaction (RT-PCR) analysis of ChAT in spinal cord. TM5484 as well as Fingolimod up-regulated ChAT expression comparing to EAE untreated mice. Expression levels of all markers are normalized to b-actin. Data are shown as the means and corresponding SEM. **P<0.01 by 1-way ANOVA and Dunnett test, n = 5–6
(EPS)

S4 Fig In spinal cord of control mice, expression of Iba-1 marker shows low levels of microglia activation, as demonstrated by none ramified branches. EAE mice show an important number of microglia activated with ramified branches (green). However, this was ameliorated by TM5484 and fingolimod. Nuclear Dapi staining (blue) confirmed the presence of viable cells.
(PDF)

S5 Fig Immunostaining. Fibrinogen deposition in spinal cord of EAE mice shows no difference in comparison to control. In addition, no changes were observed after treatment with TM5484 or fingolimod. Red asterix indicates central canal.
(PDF)
