# Supplementary figures and images for: Plasminogen Activator Inhibitor-1 Antagonist TM5484 Attenuates Demyelination and Axonal Degeneration in a Mice Model of Multiple Sclerosis
Source: PLoS One. 2015 Apr 27;10(4):e0124510. doi: 10.1371/journal.pone.0124510 (PMC4411110; doi:10.1371/journal.pone.0124510)

Supplemental Figure 4

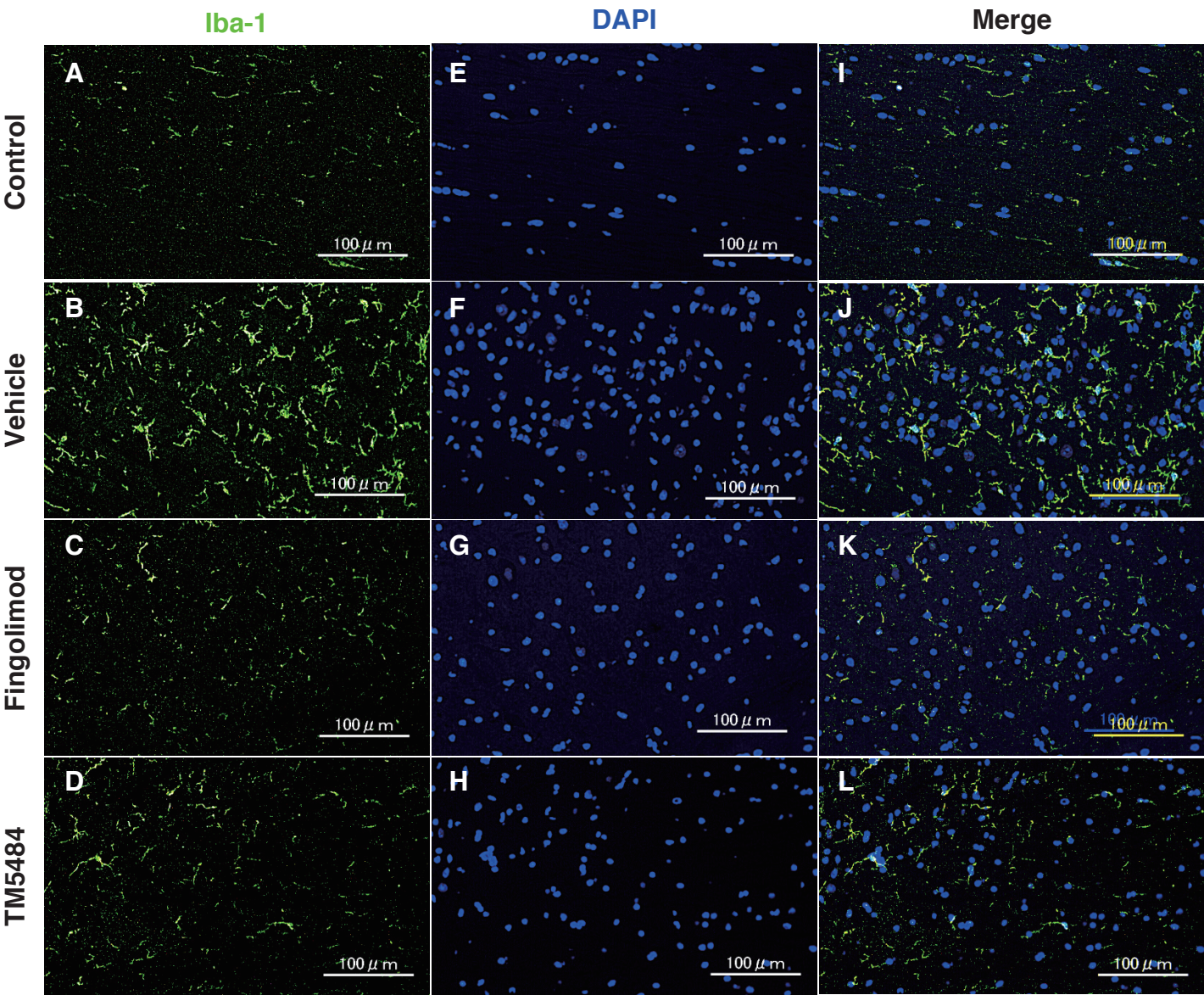

Supplement: S4 Fig — EAE mice show an important number of microglia activated with ramified branches (green). However, this was ameliorated by TM5484 and fingolimod. Nuclear Dapi staining (blue) confirmed the presence of viable cells. (PDF) [file pone.0124510.s004.pdf]

## Supplemental Figure 5

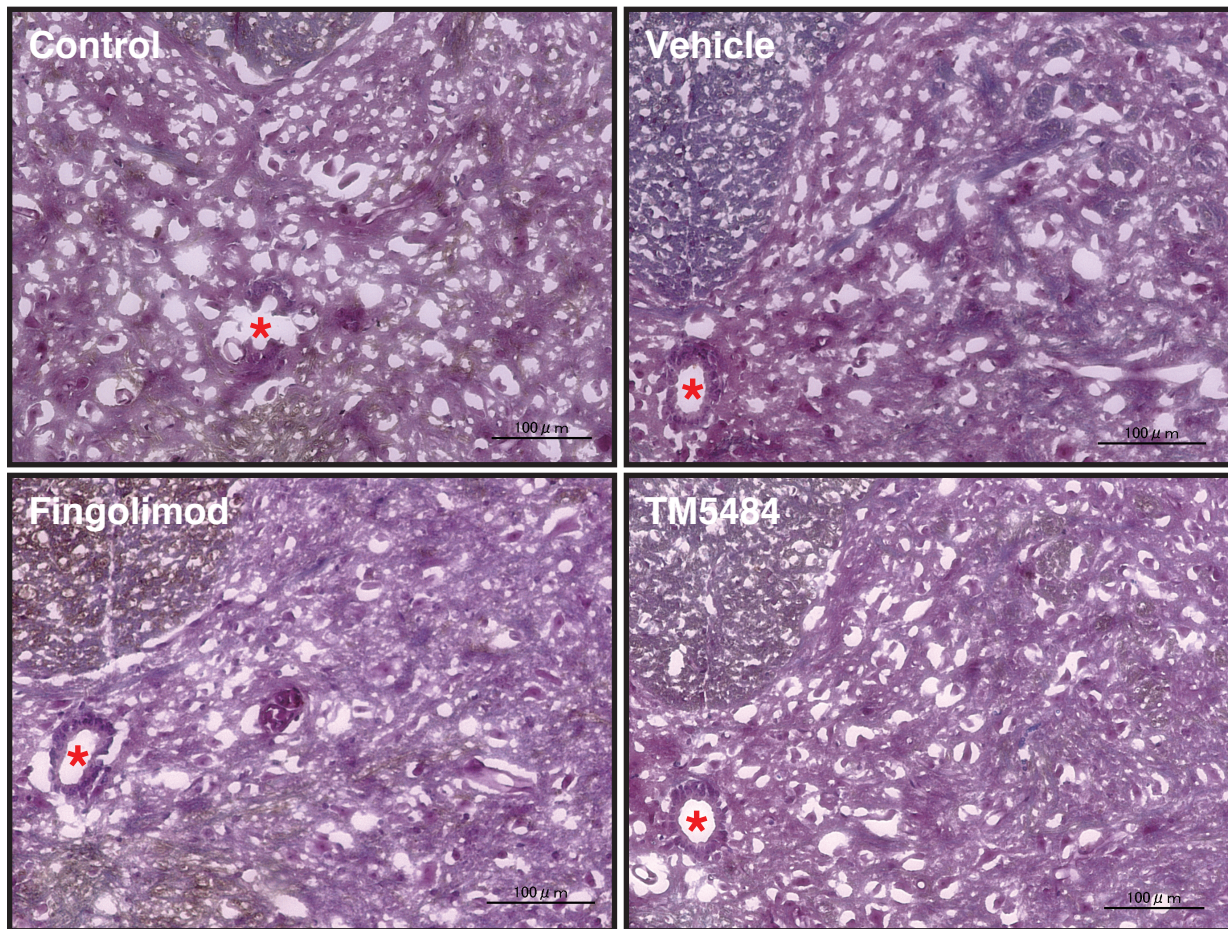

Supplement: S5 Fig — Fibrinogen deposition in spinal cord of EAE mice shows no difference in comparison to control. In addition, no changes were observed after treatment with TM5484 or fingolimod. Red asterix indicates central canal. (PDF) [file pone.0124510.s005.pdf]
